# Pharmacology activity, toxicity, and clinical trials of Erythrina genus plants (Fabaceae): an evidence-based review

**DOI:** 10.3389/fphar.2023.1281150

**Published:** 2023-11-16

**Authors:** Elis Susilawati, Jutti Levita, Yasmiwar Susilawati, Sri Adi Sumiwi

**Affiliations:** ^1^ Doctoral Program in Pharmacy, Faculty of Pharmacy, Universitas Padjadjaran, Sumedang, Indonesia; ^2^ Faculty of Pharmacy, Bhakti Kencana University, Bandung, Indonesia; ^3^ Department of Pharmacology and Clinical Pharmacy, Faculty of Pharmacy, Universitas Padjadjaran, Sumedang, Indonesia; ^4^ Department of Biology Pharmacy, Faculty of Pharmacy, Universitas Padjadjaran, Sumedang, Indonesia

**Keywords:** anti-inflammatory, Erythrina genus, flavonoids, pterocarpans, toxicity

## Abstract

The concept of using plants to alleviate diseases is always challenging. In West Java, Indonesia, a local plant, named *dadap serep* has been traditionally used to reduce blood glucose, fever, and edema, by pounding the leaves and applying them on the inflamed skin, or boiled and consumed as herbal tea. This plant belongs to the Erythrina genus, which covers approximately 120 species. The scope of this review (1943–2023) is related to the Global Development Goals, in particular Goal 3: Good Health and Wellbeing, by focusing on the pharmacology activity, toxicity, and clinical trials of Erythrina genus plants and their metabolites, e.g., pterocarpans, alkaloids, and flavonoids. Articles were searched on PubMed and ScienceDirect databases, using “Erythrina” AND “pharmacology activity” keywords, and only original articles written in English and open access were included. *In vitro* and *in vivo* studies reveal promising results, particularly for antibacterial and anticancer activities. The toxicity and clinical studies of Erythrina genus plants are limitedly reported. Considering that extensive caution should be taken when prescribing botanical drugs for patients parallelly taking a narrow therapeutic window drug, it is confirmed that no interactions of the Erythrina genus were recorded, indicating the safety of the studied plants. We, therefore, concluded that Erythrina genus plants are promising to be further explored for their effects in various signaling pathways as future plant-based drug candidates.

## 1 Introduction

Discovering herbal-based drugs is always challenging. Plants’ metabolites, e.g., flavonoids and polyphenols, have been reported for their role in representing pharmacological activities (González et al., 2011; Rosdianto et al., 2020; Stromsnes et al., 2021).

In West Java, Indonesia, a local plant, named *dadap serep*, has been traditionally used to reduce blood glucose, fever, and edema. The leaves are usually pounded and applied to the inflamed skin, or boiled and consumed as herbal tea. In order to understand the reason this plant exhibits the ability to cure diseases, we search for scientific information related to the plant. *Dadap serep*, botanical name *Erythrina subumbrans* (Hassk.) Merr.) belongs to the Erythrina genus of the family Fabaceae. The Erythrina genus covers approximately 120 species worldwide. *Erythrina subumbrans* (Hassk.) Merr.) (Fabaceae) trees are large, up to 22 m tall. The leaves are 3-pinnated, with leaf blades almost round to rhombic shape, rounded base, larger tip, with a flat edge. These plants are widely grown in the Southeast Asia region (http://ipbiotics.apps.cs.ipb.ac.id/index.php/tumbuhanObat/297). The native range of this species is from China (Yunnan) to Tropical Asia and Southwest Pacific (https://powo.science.kew.org/taxon/urn:lsid:ipni.org:names:494601-1#source-KBD).

This article aims to review the pharmacology activities, *in silico* studies, toxicity studies in animal models, and clinical trials of the Erythrina genus plants, to ensure the safety of their administration to humans.

## 2 Radical scavenging activity of the phytochemicals

The Erythrina genus of the family Fabaceae includes trees, shrubs, and herbaceous plants with orange to bright red flowers, rich in pterocarpans and alkaloids (Rukachaisirikul et al., 2007a; Phukhatmuen et al., 2021), flavonoids (Nyandoro et al., 2017; Deviani et al., 2022; Simão et al., 2022), triterpenes, steroids, alkyl trans-ferulates, proteins, saponins, and lecithin (Kumar et al., 2010), gallic and caffeic acids (Muthukrishnan et al., 2016). Pterocarpans comprise a large group of isoflavonoids and function as phytoalexins (plant antimicrobial mechanism) and are mostly found in the Leguminosae (Fabaceae) family (Jiménez-González et al., 2011). Flavonoids are low-molecular-weight polyphenolic metabolites in plants that are further classified into subgroups, e.g., flavones, flavonols, isoflavones, chalcones, and anthocyanins (Panche et al., 2016). Pterocarpans, flavonoids, and alkaloids are considered responsible for Erythrina’s pharmacology activities.

The radical scavenging activity of Erythrina genus plants has been verified by employing various methods, such as ferrous reducing antioxidant capacity (FRAC) (Afzal et al., 2022), 2,2-diphenyl-1-picrylhydrazyl (DPPH) radical (Dzoyem et al., 2014; Deviani et al., 2022; Fofana et al., 2022), and 2,2′-azino-bis (3-ethylbenzothiazoline-6-sulphonic acid) diamonium salt (ABTS) (Dzoyem et al., 2014).

The ethyl acetate fraction and lupinifolin, a flavanone isolated from *E. crista-galli* L. twigs collected in Cihideung, West Java, Indonesia, have confirmed its capability to scavenge 50% of the initial DPPH radicals with a value of 64.41 and 128.64 μg/mL, respectively, better than the other flavanones, e.g., citflavanone and lonchocarpol A. It was explained that the prenyl and the pyran in lupinifolin contribute to this capability (Deviani et al., 2022). Silver nanoparticles (Ag-NPs) synthesized from the leaf and bark extracts of *E. suberosa* Roxb. exhibited significant reducing activity towards ferric chloride solution (Afzal et al., 2022). The radical scavenging activity of *E. caffra* Thunb. (leaves) (Dzoyem et al., 2014) and *E. senegalensis* DC. (stem bark) (Fofana et al., 2022) were also delineated. Moreover, a previous *in silico* approach disclosed that the radical scavenging activity of Erythrina’s flavanone, namely, mildbone and mildbenone, is predominantly associated with the second bond dissociation enthalpy of a second hydrogen atom transfer (Anouar, 2016). Mildbone and mildbenone, polar metabolites in Erythrina, were suggested to contribute to the various biological activities of Erythrina plants (Jiménez-Cabrera et al., 2020).

## 3 Pharmacology activities

**FIGURE 1:**
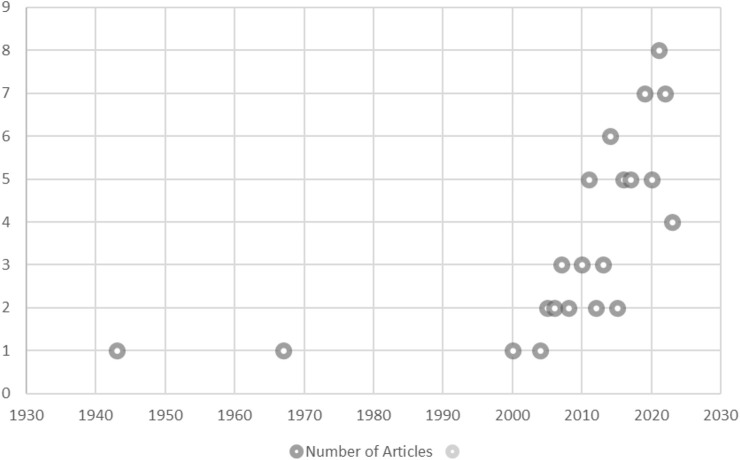
Scattered diagram of the number of reviewed articles plotted against the year of publication.

Most of the studies described Africa and Brazil as the origin locations of Erythrina plants (Fabaceae). The scope of articles was those published from 1943 to 2023 (depicted in Figure 1).

Of these articles, we found 16 species with confirmed pharmacology activities of which are:

• *E. abyssinica* Lam. ex DC. (synonym = *Carallodendron suberifera* (Welw. ex Baker) Kuntze or *Chirocalyx abyssinicus* (Lam. ex DC.) Hochst. or *Chirocalyx tomentosus* Hochst. or *E. bequaertii* De Wild. or *E. comosa* Hua or *E. eggelingii* Baker f. or *E. huillensis* Welw. ex Baker or *E. kassneri* Baker f. or *E. mossambicensis* Sim or *E. pelligera* Fenzl or *E. platyphylla* Baker f. or *Erythrina suberifera* Welw. ex Baker or *E. tomentosa* R. Br. ex A. Rich. or *Erythrina warneckei* Baker f.)
• *E. addisoniae* Hutch. & Dalziel.
• *Erythrina americana* Mill. (synonym = *Corallodendron americanum* (Mill.) Kuntze or *Corallodendron coralloides* (Moc. & Sessé ex DC.) Kuntze or *Corallodendron roseum* (A.Dietr.) Kuntze or *E. carnea* Aiton or *E. coralloides* Moc. & Sessé ex DC. or *E. enneandra* DC. or *E. fulgens* Loisel. or *E. laeta* Dehnh. or *E. rosea* A.Dietr.)
• *E. burttii* Baker f.
• *E. caffra* Thunb. (synonym = *E. constantiana* Micheli or *E. fissa* C.Presl or *E. insignis* Tod. or *Erythrina viarum* Tod.
• *E. crista-galli* L. (synonym = *Corallodendron crista-galli* (L.) Kuntze or *E. fasciculata* Benth. or *E. laurifolia* Jacq. or *E. pulcherrima* Tod. or *E. speciosa* Tod. or *Micropteryx crista-galli* (L.) Walp. or *M. fasciculata* Walp. or *M. laurifolia* Walp.)
• *E. falcata* Benth. (synonym = *Corallodendron falcatum* (Benth.) Kuntze or *E. crista-galli inermis* Speg. or *E. martii* Colla)
• *E. lysistemon* Hutch., Kew Bull. (synonym = *E. caffra mossambicensis* Baker f.)
• *E. mildbraedii* Harms (synonym = *E. altissima* A.Chev. or *E. klainei* Pierre ex Harms or *E. problematica* P.A.Duvign. & Rochez)
• *E. senegalensis* DC. (synonym = *E. guineensis* G.Don or *E. latifolia* Schumach.)
• *E. sigmoidea* Hua. (synonym = *E. dybowskii* Hua or *E. eriotricha* Harms or *E. lanata* Taub. ex Gilg or *E. sudanica* Baker f.)
• *E. suberosa* Roxb. (synonym = *Corallodendron suberosum* (Roxb.) Kuntze or *E. alba* Wight & Arn. or *E. glabrescent* (Prain) R.Parker or *E. maxima* Wight & Arn. or *E. stricta suberosa* (Roxb.) Niyomdham or *E. sublobata* Roxb. or *M. suberosa* (Roxb.) Walp. or *M. sublobata* (Roxb.) Walp.)
• *E. subumbrans* (Hassk.) Merr. (synonym = *Corallodendron lithospermum* (Blume ex Miq.) Kuntze or *E. holoserica* Kurz or *E. lithosperma* Miq. or *E. secundiflora* Hassk. or *E. sumatrana* Miq.)
• *E. variegata* Linn. (synonym = *E. indica* Lam., or *E. orientalis* (L.) Merrill, or *E. corallodendrum* var. *orientalis* L.)
• *E. velutina* Willd. (synonym = *Chirocalyx velutinus* Walp. or *Corallodendron velutinum* (Willd.) Kuntze or *E. aculeatissima* Desf. or *E. aurantiaca* Ridl. or *E. splendida* Diels)
• *E. verna* Vell. (synonym = *Corallodendron mulungu* (Mart. ex Benth.) Kuntze or *E. mulungu* Mart. ex Benth. or *E. flammea* Herzog)

The most studied activities were antibacterial and anticancer, although a few works reported anti-inflammatory, antidiabetic, estrogenic, antifungal, antidepressant, and other activities. The stem bark was the most explored and considered an active part of the Erythrina genus plants, followed by the leaves, twigs, root bark, and seeds. The pharmacology activities of the Erythrina genus plants are summarized in Supplementary Table S1.

### 3.1 Antibacterial activity

Antibacterial activity was confirmed to belong to *E. subumbrans* (Hassk.) Merr. (twigs and roots) (Phukhatmuen et al., 2021), *E. verna* Vell. (stem bark) (Simão et al., 2022), *E. suberosa* Roxb. (leaves and bark) (Afzal et al., 2022), *E. lysistemon* Hutch., Kew Bull. (stem bark) (Sadgrove et al., 2020), *E. poeppigiana* (Walpers) O.F. Cook (leaves) (Dzoyem et al., 2014). A notable antibacterial activity of plant extracts is an auspicious result since extracts contain a mixture of metabolites (Dzoyem et al., 2014). It was described previously that the prenylation of flavonoids increases the ability to penetrate the membrane cell of bacteria by building a strong lipophilic arm to the molecule. An OH group attached to the same aromatic ring as the prenyl strengthens the antibacterial activity (Sadgrove et al., 2020). Alkaloids, a group of nitrogen-containing organic compounds, were reported for their cell membrane-damaging mechanism. Alkaloids could interfere with the energy metabolisms thus inhibiting bacterial growth (Djeussi et al., 2015; Sadgrove et al., 2020; Alencar de Barros et al., 2021).

Antibacterial mechanisms of plant metabolites may include, but are not restricted to, the inhibition of bacterial cell wall synthesis, alteration of the permeability of the cell membrane permeability, and/or blocking of the bacterial synthesis of protein. Implying this, Erythrina plants, that contain flavonoids and alkaloids, may have the potential to kill Gram-positive and Gram-negative bacteria (Dzoyem et al., 2014; Djeussi et al., 2015; Sadgrove et al., 2020; Alencar de Barros et al., 2021; Phukhatmuen et al., 2021; Afzal et al., 2022; Simão et al., 2022; Yenesew et al., 2005).

### 3.2 Anticancer activity

The anticancer activity of the Erythrina genus plants has also been validated and reported in 7 articles against different cancer cell lines. The ethyl acetate subfraction of *E. senegalensis* DC. stem bark exhibited anticancer activity against U373, MCF-7, A549, SKMEL-28, and B16F10 cells, despite its lower phenolic and flavonoid content (Fofana et al., 2022). Flavanones isolated from the leaves of *E. crista galli* L. were evaluated using the *Arabidopsis thaliana* pER8: GUS reporter assay while their molecular interactions with the residues Arg394, Glu353, and His524 in the active site of human estrogen receptor R (ERR) were studied, thus revealing their anticancer activity (Ashmawy et al., 2016). The essential oils contained in the leaves of *E. variegata* Linn. blocked the proliferation of breast cancer (MDA-MB-231 and MCF-7) cells and non-cancerous mammary epithelial cells (HMLE) (Xing et al., 2019). Another *in vitro* study of *E. suberosa* Roxb. Stem bark described that two isolated metabolites, namely, 4′-methoxy licoflavanone, and alpinumisoflavone, inhibited HL-60 cell proliferation and induced apoptosis (Kumar et al., 2013). Alpinumisoflavone isolated from the stem barks of *E. lysistemon* reduced the viability and induced the apoptosis of human lung cancer cell lines H2108 and H1299 (Namkoong et al., 2011). Prenylated pterocarpans isolated from the stem bark of *E. addisoniae* Hutch. & Dalziel revealed weak to moderate toxicity towards H4IIE hepatoma cells and decreased activation of the ERK kinase (p44/p42) (Wätjen et al., 2007). Xanthoxyletin isolated from the aerial part of *E. variegata* L. induced apoptosis and cell cycle arrest in human gastric adenocarcinoma SGC-7901 cells (Rasul et al., 2011).

Taken together, apoptosis in various cancer cells may be considered the main mechanism of Erythrina metabolites. Apoptosis, identified by cell shrinkage and DNA fragmentation, is structured by a series of molecular reactions that could be stimulated extrinsically or intrinsically. Apoptosis is activated by caspases (cysteine-aspartic proteases) as effectors of the process (Hail et al., 2006). The intrinsic pathway of apoptosis is initiated when internal cellular stress occurs due to DNA damage or endoplasmic reticulum stress, which eventually activates the BCL-2 homology 3 protein. The extrinsic pathway is activated when death receptors (DR1-DR8) are induced by receptor binding or aggregation (Cavalcante et al., 2019).

### 3.3 Antidiabetic activity

Metabolites isolated from the twigs and roots of *E. subumbrans* (Hassk.) Merr. were confirmed to inhibit α-glucosidase activity and/or α-amylase activity (Phukhatmuen et al., 2021). Prenylated flavonoids isolated from the root bark of *E. mildbraedii* Harms inhibited PTP1B activity with IC_50_ values ranging from 5.3 to 42.6 mM as reported by Jang and co-workers in Korea. The assay was carried out using a human recombinant PTP1B kit. There is very little information regarding the pharmacology activity and mechanism, and no discussion has been provided since this work was presented as Notes (Jang et al., 2008). PTP1B, a tyrosine phosphatase protein, plays important contributions in intracellular signal transduction by regulating the cellular level of tyrosine phosphorylation. It downregulates insulin signaling by dephosphorylating the insulin receptor (IR) and insulin receptor substrate (IRS), thus dysfunction of this protein may lead to numerous ailments such as cancer, diabetes, and obesity (Sun et al., 2012).

An alteration in insulin signaling, predominantly in the IRS/phosphoinositide-3-kinase (PI-3K)/protein kinase B (PKB) axis, stimulates the development of insulin resistance. Insulin resistance is generally associated with obesity, which is a risk factor for type 2 diabetes mellitus (T2DM) (Wondmkun, 2020). Mature insulin is stored in granules until it is needed to be released when blood glucose levels are elevated, e.g., following a high carbohydrate intake. Several factors may also activate insulin release, e.g., amino acids, fatty acids, and hormones (Boland et al., 2017). The occurrence of insulin resistance in T2DM patients may also be caused by an abnormal synthesis of insulin, a mutation of insulin receptors, or the presence of an insulin antagonist (Al-Ishaq et al., 2019).

Interestingly, flavonoids are thought to play an important role in regulating carbohydrate hydrolysis, as well as insulin signaling and secretion (Al-Ishaq et al., 2019). It was announced that dietary intake of anthocyanins and anthocyanin-rich fruit (blueberries) was correlated with a lower risk of T2DM in both males and females (Wedick et al., 2012). A reduction in IL-6 and TNF-α was observed following the intake of different berries, suggesting an important role of these fruits in modulating the lipid profile and inflammatory cytokines in subjects with metabolic diseases (Venturi et al., 2023).

### 3.4 Anti-inflammatory activity

The anti-inflammatory activity of Erythrina plants was also reported, although limited. We found 4 articles that described the anti-inflammatory activity by various mechanisms as follows:

The fractions obtained from the dichloromethane stem bark extract of *E. verna* Vell have confirmed their inhibitory activity on nitric oxide (NO) production and blocked TNF-α activity. This *in vitro* study was performed on a murine RAW 264.7 macrophage cell line and the quantification of NO and TNF-α was done using a commercially enzyme-linked immunosorbent assay kit. In their study, Simão and others employed a bioassay-guided procedure, which resulted in two active flavonoid metabolites, namely, alpinumisoflavone and erythratidinone (Simão et al., 2022).

Previously, Machado and his co-workers studied the purification, biochemical characterization, and anti-inflammatory evaluation of a novel Kunitz trypsin inhibitor from *E. velutina* Willd. Seeds. The metabolites were purified by NH_4_(SO_4_)_2_, fractionated, and eventually underwent trypsin-sepharose affinity chromatography and an RP-HPLC. It was confirmed that the metabolites isolated from the seeds of *E. velutina* Willd. Strongly inhibited trypsin by a non-competitive mechanism, reduced leukocyte migration, decreased TNF-α release, and stimulated IFN-α and IL-12 production, thus indicating a promising anti-inflammatory activity (Machado et al., 2013).

The leaves and bark extract of *E. abyssinica* Lam. ex DC. ointments exhibited low anti-inflammatory and wound-healing properties. It was reported by Marume and the research team that wounds of animals treated with the extract ointments revealed almost similar recovery, although not significantly different, to those of animals treated with 3% oxytetracycline ointment (Marume et al., 2017).

The ethanol extract of *E. senegalensis* DC. leaves were assayed *in vivo* using egg albumin-induced paw edema in the rat model and resulted in a strong activity in stabilizing the erythrocyte membrane, and significantly decreased albumin denaturation, platelet aggregation, phospholipase A2 and protease activity (Enechi et al., 2021). Alkaloids isolated from the bark of *Erythrina crista-galli* L., purchased in September 2004 in São Paulo, Brazil, inhibit the production of nitric oxide in RAW264.7 cells (Ozawa et al., 2010). There are numerous complex mechanisms and signaling pathways related to inflammation, thus these reports may not depict the whole picture of how the botanical drugs suppress the inflammatory process. Moreover, it should be noted that different assay methods may notably affect the results.

### 3.5 Other pharmacology activities

Erythrina plants have also been explored for other activities, e.g., estrogenic activity by increasing uterus and vaginal index and keratinizing vaginal cells, inducing uterine and vaginal hypertrophy associated with endometrial proliferation (Li et al., 2019), antiviral against respiratory syncytial virus (RSV) and herpes simplex virus (HSV-2) (Mollel et al., 2022), strong antifungal activities against *Candida albicans* and *C. glabrata* strains and clinical isolates (Harley et al., 2022), Leishmanicidal activity against the promastigote forms of *L. amazonensis* (Callejon et al., 2014), anxiolytic and anti-depressant (Chu et al., 2019), bone protective effect in the tibia of ovariectomized rats (Xiao et al., 2021), antihepatotoxicity by reducing the levels of transaminases, ALP, bilirubin, and LDH to normal (Mujahid et al., 2017), retention of fear memory but did not prevent the extinction of fear memory (de Oliveira et al., 2014), Aurora kinase inhibitor (Hoang et al., 2016), antiplasmodial (Rukachaisirikul et al., 2007b; Waiganjo et al., 2020), inhibitor of neuronal nicotinic receptors (Setti-Perdigão et al., 2013).

To obtain the total active metabolites that contribute to the pharmacology activities of Erythrina genus plants, we further tabulated these metabolites and their studies on different pharmacological assays in Supplementary Table S2.

## 4 *In silico* study

Regrettably, phenolic metabolites of the Erythrina genus plants have been very limitedly studied for their interaction with various proteins. However, the limited studies are described below.

Phenolic metabolites of *Erythrina × neillii* (a cross-hybrid between *E. herbacea* L. and *E. humeana* Spreng), were reported for their interaction with Arg136, Met34, and Gly139 of heme oxygenase-1 (HO-1), a macromolecule that plays a role in cellular protection and oxidative catabolism of heme. Stimulation of HO-1 may reduce cardiovascular disease (Gabr et al., 2019). Phaseollin of *E. variegata* L. built interaction with significant fitness score and hydrogen bonds with Tyr354 and Ser499 residues in both COX-1 and COX-2, which is considered a potent analgesic candidate (Uddin et al., 2014). Tyr354 is one of the key residues constituting the active site of human COX-1, along with Arg119, Tyr384, Ile433, His512, Phe517, and Ile522 (Dhanjal et al., 2015). Polyphenolic metabolites isolated from *E. crista-galli* L. could interact with Arg394, Glu353, and His524 residues of the human estrogen receptor R (ERR), thus revealing a promising phytoestrogenic activity (Ashmawy et al., 2016). Moreover, diprenylgenistein and phaseollin isolated from the twigs of *E. crista-galli* L. showed the highest binding affinity towards CDK2 protein, revealing its anticancer activity (Imanuddin et al., 2023). Cyclin-dependent kinases (CDKs) are proteins that regulate the transition of cell cycle phases. Cyclin E binds and activates CDK2 to promote S-phase entry and progression. Cyclin E/CDK2 complex eventually phosphorylates various substrates to control essential cellular processes (Fagundes and Teixeira, 2021).

There is no report on the *in silico* study of pterocarpans of the Erythrina genus plants, thus unlatching the chance to be further investigated. However, a few articles reported *in silico* studies of pterocarpans from other plants.

One article reported that pterocarpans isolated from *Desmodium gangeticum* could interact with tyrosine phosphate kinase similarly to the protein’s inhibitors (Meena et al., 2022). Protein kinases are responsible for the phosphorylation process to activate the function of other proteins. Inhibitors of kinases interact in different modes depending on the specific binding sites and conformational aspects of the enzyme, which are determined by two conditions, e.g., (1) the Asp-Phe-Gly (DFG) motif, as part of the activation loop, and (2) the αC-helix. Most kinase inhibitors interact in the active form (conformational state: DFG-in, αC-helix-in), and are classified as type I inhibitors. Type II inhibitors attach to the inactive state (conformational state: DFG-out, αC-helix-out) (Abdelbaky et al., 2021). It is interesting to explore the binding modes of pterocarpans to kinases and whether these compounds are categorized as type I or type II inhibitors.

Pterocarpans isolated from *Indigofera aspalathoides* were reported could interact with Ser530 and Tyr385 in COX-1 and COX-2 binding pockets. The interaction with Ser530 is believed to be important in inhibiting the catalytic process of these enzymes (Selvam et al., 2004). In the biosynthesis of prostaglandin H2 (PGH2), the substrate of COX, namely, arachidonic acid, has to enter the cyclooxygenase narrow channel with its carboxylate group attaches (by building hydrogen bonds) to Arg120 and Tyr355, two residues located at the channel opening, and its ω-end occupies in a hydrophobic cavity at upwards position of Ser530 (Malkowski et al., 2000; Vecchio et al., 2010). It has been confirmed that acetylation of Ser530 by acetylsalicylic acid could inhibit the synthesis of prostaglandins (Giménez-Bastida et al., 2019).

Another *in silico* study confirmed that pterocarpans contained in *Sophora flavescens* interact with neuraminidase by occupying a binding pocket adjacent to the active site. The methanol extract of this plant at 30 ppm reduced neuraminidase activity by 90% (Ryu et al., 2008).

## 5 Toxicity studies

In fact, there are only very limited toxicity studies on Erythrina genus plants.

An acute toxicity study of *E. senegalensis* DC. stem extract in mice (*Mus musculus*) with doses of 1,250 to 12,500 mg/kg body weight (BW) did not induce mortality or significant behavioral changes. In a sub-chronic toxicity study in rats (*Rattus norvegicus*), daily oral administration of *E. senegalensis* DC. stem extract at the dose of 600 mg/kg BW resulted in a significant elevation in the relative BW but no significant alterations in the hematological parameters, suggesting its safety (Atsamo et al., 2011).

Chronic oral toxicity studies of *E. mulungu* Mart. ex Benth. Mart. ex Benth. (synonym = *E. verna* Vell. or *Corallodendron mulungu* (Mart. ex Benth.) Kuntze or *E. flammea* Herzog) stem bark extract in mice (*Mus musculus*) at a dose of 1,000 mg/kg BW resulted in alterations of biochemical parameters of the liver and cardiovascular functions. Hematological analysis indicated significant decreases in the red blood cells. Oral daily doses of 500 and 1,000 mg BW of *E. mulungu* Mart. ex Benth. Stem bark extract increased the relative weights of the liver and heart and serum malondialdehyde levels. Significant histopathological changes in myocardial and degeneration of hepatocytes also occurred (Adeneye, 2014).

A sub-chronic toxicity study of the methanol extract of *E. variegata* Linn. leaves on male Wistar rats (*Rattus norvegicus*) revealed slight changes in hematological parameters but were within the normal range, except for BUN and SGPT. Histopathological examination indicated increased damage to the liver and kidney cells, however, doses of 250, 500, and 1,000 mg/kg BW did not cause toxicity in animal models (Herlina et al., 2017). Table 1 tabulates the toxicity studies of the Erythrina plants.

**TABLE 1:** Toxicity studies of the Erythrina genus plants.

Plant extract	Toxicity study	Animal model	Results and doses	Reference
*E*. *senegalensis* DC. aqueous stem extract was prepared from the stem bark (collected in Dschang, West region of Cameroon, Africa, in February 2007)	Acute	Mice (*Mus musculus*)	• No mortality	Atsamo et al. (2011)
			• No behavioral changes at doses of 1,250–12,500 mg/kg BW which indicated the safety of the plant extract	
	Sub-chronic	Rats (*Rattus norvegicus*)	• A significant elevation in the relative BW	
			• No significant alterations in the hematological parameters	
			• Doses of ≥600 mg/kg BW did not cause mortality which indicated the safety of the plant extract	
*E. mulungu* Mart. ex Benth. Stem bark extract	Chronic	Mice (*Mus musculus*)	• Alterations of biochemical parameters of the liver and cardiovascular functions at a dose of 1,000 mg/kg BW	Adeneye (2014)
			• Alterations of hematological analysis (significant decreases in the red blood cells)	
			• Increases the relative weights of the liver and heart at doses of 500 mg/kg BW and 1,000 mg/kg BW	
			• Increases the serum MDA levels and lipid peroxidation marker at doses of 500 mg/kg BW and 1,000 mg/kg BW	
			• Significantly changes the histopathological of myocardial and degeneration of hepatocytes	
			• A dose of 1,000 mg/kg BW did not cause mortality which indicated the safety of the plant extract	
*E*. *variegata* Linn. methanolic leaves extract was prepared from the leaves (collected in Sumedang, West Java, Indonesia)	Sub-chronic	Male Wistar rats (*Rattus norvegicus*)	• Slight changes in hematological parameters but were within the normal ranges	Herlina et al. (2017)
			• Increase of BUN, SGOT, and SGPT	
			• Higher doses increased damage to the liver and kidney cells	
			• Doses of 250, 500, and 1,000 mg/kg BW did not cause toxicity in animal models	
			• A dose of 1,000 mg/kg BW did not cause mortality which indicated the safety of the plant extract	

The toxicity of the metabolites contained in the Erythrina genus plants was not commonly reported. However, an article written by Unna and Greslin explained that erythroidine, an alkaloid isolated from *Erythrina americana* Mill., and other alkaloids, namely, 9-erythroidine, erythramine, erythraline, and erythratine revealed a curarizing effect similarly to that of the crude seed extracts (Unna and Greslin, 1943). It was pointed out that the curarizing effect of a drug changes the electrical activity of the cerebral cortex chiefly due to hypoxia and hypotension (Skorobogatov, 1967). The other major metabolites such as flavonoids and pterocarpans have been proven as safe.

## 6 Clinical study

There are very limited studies on the efficacy and safety of the Erythrina genus plants in humans. We found only 2 articles, both using *E. mulungu* Mart. ex Benth. (synonym = *E. verna* Vell. or *Corallodendron mulungu* (Mart. ex Benth.) Kuntze or *E. flammea* Herzog) as an antianxiety in patients who underwent dental surgery.

**Study 1.** A randomized, triple-blind, clinical control trial was performed on 200 patients who underwent oral surgery of their third mandibular molars (consort number NCT02065843). The patients were treated with either *Passiflora incarnata* (500 mg), *E. mulungu* Mart. ex Benth. (500 mg), or midazolam (15 mg) orally 60 min prior to the surgery. The anxiety level of the patients was analyzed using questionnaires and measurements of heart rate, blood pressure, and oxygen saturation (SpO2). It was reported that *P. incarnata* revealed a similar effect to midazolam but differed from placebo and *E. mulungu* Mart. ex Benth., indicating that *E. mulungu* could not be used to reduce the anxiety of the patients (da Cunha et al., 2021).

**Study 2.** A similar randomized, double-blind, crossover study was performed on 30 healthy adult patients who underwent bilateral surgery of asymptomatic, impacted mandibular third molars (consort number NCT01948622). The patients were treated with either *E. mulungu* Mart. ex Benth. (500 mg) or a placebo (500 mg) orally 60 min before the surgery. To avoid pain and swelling after surgery, the patients were given a single dose of intra-muscular dexamethasone and an antiseptic mouthwash containing chlorhexidine gluconate 30 min before surgery. The level of anxiety was analyzed using questionnaires and measurements of blood pressure, heart rate, and oxygen saturation. It was reported that *E. mulungu* Mart. ex Benth. had shown an anxiolytic effect without significant changes in physiological parameters (Silveira-Souto et al., 2014).

## 7 Botanical drug-drug interaction

Extensive caution should be considered when prescribing botanical drugs for patients parallelly taking a narrow therapeutic window drug. The botanical drugs may dangerously alter the main drug or cause ineffectiveness. Taking this into consideration, we studied the Herb-Drug Interaction Chart (Mills and Bone, 2005; ESCOP, 2023; Standard Process, 2023) and found that no interactions of the Erythrina genus were recorded, indicating the safety of the studied plants.

## 8 Conclusion

There are 16 of the 120 species of the Erythrina genus plants with confirmed pharmacological activities. Antibacterial and anticancer were the most studied activities, although a few works reported anti-inflammatory, antidiabetic, estrogenic, antifungal, antidepressant, and other activities. The extracts of the Erythrina genus have revealed anticancer activity against human glioblastoma astrocytoma (U-373), human breast cancer cell line with estrogen, progesterone, and glucocorticoid receptors (MCF-7), human epithelial breast cancer (MDA-MB-231), human melanoma (SKMEL-28), murine melanoma (B16F10), human promyelocytic leukemia (HL-60), lung cancer (H2108, H1299, H411E), human adenocarcinoma alveolar basal epithelial (A549), and human gastric cancer (SGC-7901) cells. Studies of the effect of these plant extracts on non-cancerous cells are limited, nevertheless, a study reported that the Erythrina plant extract did not show cytotoxicity on HMLE (immortalized human mammary epithelial), a non-cancerous cell.

The stem bark was the most explored and considered the main active part of the Erythrina genus plants. Sixty-three metabolites have been isolated from various parts of the plants, comprised of pterocarpans, flavonoids, alkaloids, triterpenoids, glycosides, steroids, chromenes, coumarins, and carboxylic acids. The most abundant classes are pterocarpans and flavonoids.

There are very limited toxicity studies on Erythrina genus plants, among those are *E. senegalensis* DC., *E. mulungu* Mart. ex Benth. (synonym = *E. verna* Vell. or *Corallodendron mulungu* (Mart. ex Benth.) Kuntze or *E. flammea* Herzog), and *E. variegata* Linn. All these studies indicated the safety of the higher doses of the plant extracts in animal models. The toxicity of the metabolites contained in the Erythrina genus plants was not commonly reported. However, alkaloids isolated from *E. americana* Mill. namely, erythroidine, 9-erythroidine, erythramine, erythraline, and erythratine were reported for their curare-like effect on frogs.

There are very limited human studies on Erythrina genus plants. Only one species, *E. mulungu* Mart. ex Benth. has been reported as a weak anxiolytic in dental surgery in randomized, controlled clinical trials. It should be noted that the selected articles have employed different experimental designs.

Most of the pharmacological studies were carried out on plant extracts, not as nutraceuticals or supplements, or pure metabolites, and almost all studies reported significant statistical analysis results. Not all articles described their procedures in detail, thus affecting the reliability of the studies and limiting our interpretations. Nonetheless, we feel optimistic that the Erythrina genus plants are promising to be further explored for their effects in various signaling pathways as future plant-based drug candidates. We are certain this review has unraveled comprehensive perspectives of Erythrina genus plants thus providing beneficial insights into plant-based drug discovery.
