# Supplementary material for: Pharmacology activity, toxicity, and clinical trials of Erythrina genus plants (Fabaceae): an evidence-based review
Source: Front Pharmacol. 2023 Nov 16;14:1281150. doi: 10.3389/fphar.2023.1281150 (PMC10690608; doi:10.3389/fphar.2023.1281150)
Supplement: Supplementary file 1 [file Table1.docx]

**Table 1**. Pharmacology Activities of the Erythrina Plants

| Name of the Erythrina Plant (family), Collected in, Time of Collection. | Part of the Plant and Solvent Used for Extraction | Type of Study | Pharmacology Activity | Range of Dose or Concentration and Duration | Control Used | Results | Reference |
| --- | --- | --- | --- | --- | --- | --- | --- |
| *E. subumbrans* (Hassk.) Merr. (Fabaceae) twigs and roots were collected in Doi Tung, Chiang Rai, Thailand (20°20′ 27.00″ N and 99° 50′ 2.39″ E), in 2019. | The twigs and roots were extracted with ethyl acetate (EtOAc) for 3 days at room temperature.  The root extract was fractionated by quick column chromatography (QCC) on silica gel with a gradient of hexanes-acetone to give 7 fractions.  The twig extract was subjected to QCC on silica gel with a gradient of hexanes-EtOAc to obtain 8 fractions. | *In vitro* using α-glucosidase and α-amylase screening kit. | Antidiabetic | Not described | Acarbose, voglibose, and quercetin | Twenty-eight metabolites (14 pterocarpans, 5 flavanones, 3 flavones, 3 isoflavones, and 3 phenolic derivatives) have been isolated from the twigs and root extracts of *E. subumbrans*.  Metabolites 3, 8, 9, and 22 strongly inhibited α-glucosidase activity with IC_50_ values < 30 μM.  Metabolite 2 inhibited α-amylase activity with an IC_50_ value of 67.6 ± 1.12 μM.  Statistical analysis was not described. | [5] |
|  |  | *In vitro* by determining the glucose consumption assay on 3T3-L1 embryonic cells. |  |  | Metformin | Metabolite **2** strongly inhibited *α*-glucosidase and promoted glucose consumption activity in 3T3-L1 cells, without causing toxicity to cells.  Statistical analysis was not described. |  |
|  |  | *In vitro* on:   - *Enterococcus faecalis* - *Micrococcus luteus* - Methicillin-resistant *S. aureus* - *S. aureus* - *Streptococcus pyogenes* - *Shigella flexneri* - *P. aeruginosa* - *S. typhi* - *S. typhimurium* - Fungi *Candida albicans* | Antibacterial and antifungal | Not described | Not described | Metabolite **9** showed the best activities against Gram-positive bacteria and fungi with MIC values ranging from 2-4 μg/mL, whereas compound **10** was less active with MIC values ranging from 8-64 μg/mL.  Statistical analysis was not described. |  |
| *E. verna* Vell. (Fabaceae) stem barks were collected in São José dos Calçados, Brazil, in September 2014. | The stem bark was extracted with dichloromethane in a soxhlet apparatus for 4 hours and percolated with a hydrochloric acid solution.  The obtained solution was added with ammonium hydroxide until pH 10 and extracted with dichloromethane. The dichloromethane extract was also fractionated by adsorption chromatography using silica-gel 60 (Merck) and binary solvent mixtures and resulted in 9 fractions.  From the fraction F4, two isoflavones were isolated: alpinumisoflavone and erysenegalensein M, whereas erythratidinone was obtained from the alkaloidal fraction obtained by acid-base extraction. | *In vitro* on:   - avirulent   *Mycobacterium bovis*Bacillus Calmette-Guérin (BCG) Moreau strain   - *Mycobacterium tuberculosis*H37Rv - Highly virulent Mtb Beijing strain M299 isolated from TB patients in Mozambique | Antibacterial | 100, 20, 4, and 0.8 µg/mL | Rifampicin | Fractions 4, 6, and 9 showed significant growth inhibitory capacity against mycobacterial strains Mbv BCG and Mtb H37Rv. | [7] |
|  |  | *In vitro* on LPS-stimulated RAW 264.7 macrophages | Anti-inflammatory | Not described | L-NMMA (a non-selective nitric oxide synthase inhibitor) | Fractions 1-9 inhibited NO production.  Fractions 2, 4, and 5 inhibited TNF-α activity.  Alkaloid erythratidinone and isoflavone erysenegalensein M reduced the production of both pro-inflammatory mediators (p < 0.001).  Data were analyzed by one-way ANOVA and Tukey test for multiple range tests, using GraphPad Prism 4 software to assess statistical significance between groups. |  |
| *E. suberosa* Roxb. (Fabaceae) fresh leaves and bark were collected in Bagh-e-Jinnah Lahore, Pakistan. | The leaves and bark were dried at room temperature and extracted with methanol. | *In vitro* on:   - *E. coli* - *P. aeruginosa* - *S. aureus* | Antibacterial using agar well diffusion method. | 15, 31, 62, 125, 250 and 500 µg/mL | Not described | The bark showed effective MIC at 63, 250, and 125 µg/mL concentrations against *E. coli, P. aeruginosa*, and *S. aureus*, respectively, with a significant value (p ≤ 0.05).  The leaves showed effective MIC at 250 µg/mL against *E. coli* and 125 µg/mL against S. aureus and *P. aeruginosa,* with a significant value (p ≤ 0.05).  Data were analyzed by one-way ANOVA and followed by post-hoc Tukey test using the level of significance p ≤ 0.05. The difference between specific groups was determined using LSD (least significant differences). | [13] |
| *E. caffra* Thunb. (Fabaceae) leaves were collected in the Pretoria National Botanical Garden in South Africa. | The dried leaves were extracted with acetone three times. | *In vitro* on three Gram-positive bacteria:   - *S. aureus* - *E. faecalis* - *Bacillus cereus*   and three Gram-negative bacteria:   - *E. coli* - *P. aeruginosa* - *S. typhimurium* | Antibacterial | Not described | Not described | Antibacterial activity against *E. faecalis* (MIC of 80 μg/ml), but the significance (p-value) was not described.  Statistical analysis was performed using one-way ANOVA and results were compared using Fisher's LSD at a 5% significance level. | [14] |
| The stem bark of *E. senegalensis* DC. (Fabaceae) was collected in an area located 17 km (UTM: X = 30P03434582; Y = 1239658) from Bobo-Dioulasso (Burkina Faso), Africa, in January 2017. | The stem bark was extracted with CH_2_Cl_2_/methanol (1:1) for 24-hour maceration.  The CH_2_Cl_2_ extract was fractionated in an open chromatography column on silica gel with successive elutions using n-hexane, ethyl acetate, and acetone. | *In vitro* on U373 (human glioblastoma astrocytoma), MCF-7 (human adenocarcinoma), A549 (human adenocarcinoma alveolar basal epithelial), SKMEL-28 (human melanoma), and B16F10 (murine melanoma) cells | Antiproliferative | Not described | Not described | The pro- or antioxidant effects observed did not correlate with their IC_50_ concentrations against five cancer cell lines determined by MTT assay.  The CH_2_Cl_2_ extract and its ethyl acetate (EtOAc) subfraction appeared more potent although they harbored lower pro- or antioxidant effects.  At equipotent concentration, both extracts induced ER- and mitochondria-derived vacuoles observed by fluorescent microscopy that further led to non-apoptotic cell death.  Statistical analysis was not described. | [15] |
| The stem bark of *E. lysistemon* Hutch., Kew Bull. (Fabaceae) was collected in the Johannesburg Faraday muthi market, South Africa. | The stem bark was extracted with either methanol or dichloromethane for 48 hours at room temperature. | *In vitro* on Gram-negative strains:   - *E. coli* - *P. aeruginosa*   and Gram-positive strains:   - *S. aureus* - *S. epidermidis* - *B. cereus* | Antibacterial | Not described | Ciprofloxacin | The metabolites isolated from the stem bark showed antibacterial activity with MIC values ranging from 1–600 ppm, with no obvious pattern of selectivity for Gram-types.  Statistical analysis was not described. | [18] |
| *E. poeppigiana (*Walpers) O.F. Cook (Fabaceae) seeds were purchased from ArboCenter company seed trade, (lot: 00316, 2012 crop) | Seed teguments were manually removed, cotyledons were ground, defatted with hexane, and used in a classical protein extraction process.  The seed flour was extracted with 100 mM sodium phosphate buffer, pH 7.6 (1:10; w/v) overnight at 4 °C. | *In vitro* on:   - *E. coli* - *Enterobacter aerogenes* - *Enterobacter cloacae* - *K. pneumoniae* - *Serratia marcescens* - *Acinetobacter baumannii* - *Salmonella enterica* - *Proteus vulgaris* - *P. aeruginosa* - *S. aureus* - *S. haemolyticus* - *S. saprophyticus* | Antibacterial |  | Ciprofloxacin and vancomycin | Antibacterial activity with MIC of 5–10 µM and MBC of 10 µM for *Enterobacter aerogenes, Enterobacter cloacae, K. pneumoniae, S. aureus*, and *S. haemolyticus* (p < 0.0001).  Statistical analysis was performed using GraphPad Prism. All data were submitted to the Shapiro–Wilk test to check for normal distribution. The data concerning the biofilm assays were analyzed by one-way ANOVA with Tukey’s multiple comparison test, with a 5% significance level. | [19] |
| The leaves and bark of *E. sigmoidea* Hua. (Fabaceae) were collected in Bangangté West Region of Cameroon, Africa, in April 2013. | Leaves and bark | *In vitro* on:   - *E. coli* - *Enterobacter cloacae* - *K. pneumoniae* - *Providencia stuartii* - *P. aeruginosa* | Antibacterial | Not described | Not described | The crude extracts from the leaves and bark inhibit the growth of 96.3% of the tested bacteria.  Statistical analysis was not described. | [20] |
| The stem bark of *E. burttii* Baker f. (Fabaceae) was collected near Emali town, on the Nairobi–Mombasa road, Kenya, Africa, in March 2001. | Stem bark | *In vitro* | Antibacterial | Not described | Not described | The chloroform extract showed antifungal and antibacterial activities using the disk diffusion method.  Statistical analysis was not described. | [21] |
| *E. crista-galli* L. (Fabaceae) fresh leaves were collected in El-Giza Zoo garden, Giza. Egypt, in April 2014. | The fresh leaves were extracted with double-distilled water for 6 hours. | *In vitro* using *Arabidopsis thaliana* pER8: GUS reporter assay and the proliferation-enhancing activity of MCF-7 cells. | Phytoestrogenic and cytoprotective activities | Not described | 17β-estradiol | The minimum active concentration (MAC) of 17β-estradiol was 2.5 nM.  Fractions I and II exhibited strong phytoestrogenic activity with MAC values < 6.25 ppm. Aqueous and aqueous methanol extracts showed less activity.  Statistical analysis was not described. | [22] |
| *E. corallodendron* L. (Fabaceae) leaves were collected from Zhangzhou, Fujian, China, in August 2018. | Essential oil was obtained from the fresh leaves by a hydro-distillation method using a Clevenger apparatus for 3 hours. | *In vitro* on MDA-MB-231, MCF-7, and HMLE cells | Anticancer | 0.25, 0.5, 1, 2, 4, 8, and 16 μg/mL | Doxorubicin and capecitabine | The essential oils inhibit the proliferation of breast cancer (MDA-MB-231 and MCF-7) cells and non-cancerous mammary epithelial cells (HMLE).  The essential oils inhibit migration and invasion of breast cancer cells.  Concentrations of 1, 2, 4, 8, and 16 μg/mL significantly inhibit migration and invasion of breast cancer cells (p < 0.05).  Data were analyzed using SPSS 16.0 and one-way ANOVA was used for multiple comparisons. | [23] |
| *E. suberosa* Roxb. (Fabaceae) stem bark was collected in India. | Stem bark | *In vitro* on HL-60 cells | Anticancer | Not applicable | Not described | Four active metabolites hydroxyerysotrine, 4′-methoxy licoflavanone (MLF), alpinumisoflavone (AIF), and wighteone have been isolated.  Both MLF and AIF inhibited HL-60 cell proliferation, induced apoptosis, and significantly inhibited nuclear transcription factor NF-kappaB and STAT (signal transducer and activator of transcription) signaling pathway (p < 0.05).  Data were analyzed by one-way ANOVA and followed by post-hoc Tukey test using the level of significance p ≤ 0.05. | [24] |
| The stem bark of *E. lysistemon* Hutch., Kew Bull. (Fabaceae) was collected in Buea, Cameroon, Africa, in July 2003. | The ground stem bark was extracted with methanol at room temperature for two weeks. | *In vitro* on human lung cancer cell lines H2108 and H1299 | Anticancer | 0, 5, 15, 30 mM for 24 hours | Not described | Alpinumisoflavone isolated from the stem bark inhibits viability and induces the apoptosis of human lung cancer cell lines H2108 and H1299.  Statistical analysis was not described. | [25] |
| *E. addisoniae* Hutch. & Dalziel (Fabaceae) | Stem bark | *In vitro* on H4IIE hepatoma cells | Anticancer |  | Not described | Prenylated pterocarpans isolated from the stem bark revealed weak to moderate toxicity towards H4IIE hepatoma cells.  Neorautenol and phaseollin did not affect the NF-kappaB signaling but reduced the activation of the ERK kinase (p44/p42).  Statistical analysis was not described. | [26] |
| *E. variegata* L. (Fabaceae) leaves were collected in China. | The leaves were extracted by a Soxhlet apparatus with alcohol for more than 12 cycles. | *In vitro* on human gastric adenocarcinoma SGC-7901 cells | Anticancer | 30, 60, 125, 250, 500, and 1000 µM of xanthoxyletin | Not described | Xanthoxyletin isolated from the leaves induced apoptosis and cell cycle arrest in SGC-7901 cells (p < 0.05).  Statistical comparisons were made by Student’s t-test and p < 0.05 was considered statistically significant. | [27] |
| The root bark of *E. mildbraedii* Harms (Fabaceae) was collected in July 1997, in Buea, Southwest Province, Cameroon, Africa. | The root bark was extracted with ethyl acetate at room temperature for two weeks. | *In vitro* using PTP1B (human, recombinant) kit (BIOMOL) | Antidiabetic and antiobesity | Not described | Not described | Prenylated flavonoids isolated from the root bark inhibited PTP1B activity with IC50 values ranging from 5.3 to 42.6 mM.  Statistical analysis was not described. | [31] |
| *E. velutina* Willd. (Fabaceae) The location where the seeds were obtained, was not described. | The seeds were peeled and cotyledons were ground at 6°C and the flour was homogenized. | *In vitro* | Anti-inflammatory | Ranged between 5 to 50 x 10^-8^ mol/L | Not described | Inhibits trypsin with IC_50_ of 2.2×10^-8^ mol/L and with Ki of 1.0×10^-8^ mol/L by a non-competitive mechanism, and inhibits factor Xa and neutrophil elastase, but does not inhibit thrombin, chymotrypsin or peptidase 3.  Differences between groups were compared by using ANOVA and Tukey Test. Differences were considered significant when p-value < 0.05. Statistical data were analyzed by GraphPad Prism 5.0 software. | [38] |
| The leaves and bark of *E. abyssinica* Lam. ex DC. (Fabaceae) were collected in Karoi, Mashonaland West Province (16°49′44.1″S 29°41′19.8″E) | The leaves and bark were extracted (1:20 w/v) with 50% aqueous methanol in an ultrasonic bath for 1 hour. | *Ex vivo* in wounded albino female BALB/c mice *(Mus musculus*) | Wound healing/anti-inflammatory | Given as ointments | White soft paraffin and 3% oxytetracycline ointment | The leaves and bark extract ointments exhibited low wound healing properties (p > 0.05).  Data on wound contraction percentage area changes and crude protein content across the groups was analyzed using regression models, ANOVA, and post-hoc LSD at *p* = 0.05 significance level. The SPSS version 21 was used for all data analysis. | [39] |
| The fresh leaves of *E. senegalensis* DC. (Fabaceae) were collected in Ugwu-Awgbu village, Orumba North of Anambra-Nigeria. | The leaves were extracted with ethanol 95% for 72 hours. | *In vitro* on human red blood cell (HRBC) membrane | Anti-inflammatory | 200-1000 mg/mL | Aspirin | The extract stabilized the HRBC membrane and significantly suppressed albumin denaturation, platelet aggregation, phospholipase A2, and protease activity (p < 0.05).  Data were examined with one-way and two-way ANOVA using version 23.0 of SPSS. The difference in mean values was deemed significant at p < 0.05. | [40] |
|  |  | *In vivo* in mice *(Mus musculus*) |  | 100, 200 and 350 mg/kg BW |  | The extract significantly (p < 0.05) decreased rat paw edema formation in a time-dependent (0.5-5 hours) manner.  Data were examined with one-way and two-way ANOVA using version 23.0 of SPSS. The difference in mean values was deemed significant at p < 0.05. |  |
| The bark of *E. crista-galli* L. (Fabaceae) was purchased in September 2004 in São Paulo, Brazil. | The bark was extracted with methanol and successively partitioned between petroleum ether, EtOAc, and 3% aqueous tartaric acid. Water-soluble materials were adjusted to pH 10 with sodium carbonate and partitioned with chloroform, EtOAc, and n-butanol, successively | *In vitro* on LPS-induced RAW264.7 cells | Anti-inflammatory | 1.56, 6.25, 25 mg/mL | Not described | Erythraline, erythrinine, and an indole alkaloid hypaphorine inhibited LPS-induced nitric oxide production with IC_50_ values of 8.8, 3.4, and 11.2 mg/ml, respectively.  Statistical analysis was not described. | [41] |
| The stem bark of *E. variegata* L. (Fabaceae) was collected in Shenzhen, China, in May 2003. | Genistein and 8-prenyl genistein (8-PG) isolated from the stem bark extract | *In vivo* on 1-month-old immature female CD-1 mice *(Mus musculus*) | Oestrogenic activity | 75 and 150 mg/kg BW of genistein and 8-PG | Vehicle | Genistein and 8-PG increased the uterus index and vagina index compared to untreated control (p < 0.05).  Accordingly, both genistein and 8-PG made vaginal cells keratinized and induced uterine and vaginal hypertrophy associated with endometrial proliferation.  Statistical analyses were performed using GraphPad Prism version 4.0. Intergroup differences were analyzed by one-way ANOVA and followed by Tukey’s multiple comparison test. Differences with p < 0.05 were considered statistically significant. | [42] |
| *E. abyssinica* Lam. ex DC. (Fabaceae) plant samples were collected in the Kagera region, Tanzania. | The plant part was not described in detail. The plant samples were macerated in n-hexane twice for 48 hours at room temperature, filtered, and the residue subjected to successive maceration with ethyl acetate followed by distilled water under conditions identical to the n-hexane maceration. | *In vitro* on human laryngeal epidermoid carcinoma HEp-2 cells and African green monkey kidney (GMK AH1) cells | Antiviral against RSV A2, HSV-2, and HPIV-2 | 0.8–500 μg/mL for n-hexane and ethyl acetate extracts or 1.6–1000 μg/mL for water extract | Not described | The n-hexane and ethyl acetate extracts were not active.  The water extract inhibited the infectivity of RSV and HSV-2 but not HPIV-2.  Statistical analysis was performed using GraphPad Prism version 9.0.1. IC_50_ and CC_50_ values were calculated using the sigmoidal dose-response equation (with variable slope). An unpaired student t-test was used for the analysis of yield reduction and virus inactivation of both water extracts and ethanol precipitates. *P values* ≤ 0.05*, ≤0.01** and ≤0.005*** were considered significant. | [43] |
| *E. senegalensis* DC. (Fabaceae) stem bark was collected in Ejura, Ashanti Region of Ghana, in May 2020. | The dried material was Soxhlet-extracted with 70% ethanol for 24 hours. | *In vitro* on fungal strains:   - *C*. *albicans* - SC5314 strains - *C*. *glabrata*   and on *C. albicans* and *C. glabrata* clinical isolates that were resistant to either nystatin or azole antifungals | Antifungal | 1000–1.95 μg/mL | Voriconazole and nystatin at 128–0.125 μg/mL | Strong antifungal activities against *C. albicans* and SC5314 strains, and *C. glabrata* (MIC values 3.91-31.25 μg/mL and MFCs 62.5-250 μg/mL).  Potent antifungal activity (MIC = 4-64 μg/mL) against *C. albicans* and *C. glabrata* clinical isolates that were resistant to either nystatin or azole antifungals.  Statistical analysis was not described. | [44] |
| *E. verna* Vell. (Fabaceae) bark was acquired commercially by a registered supplier of plant matter in Brazil. | The bark was extracted by percolation with ethanol and erythraline, the major spirocyclic alkaloid, was isolated. | *In vitro* on promastigote forms obtained from infected mice | Leishmanicidal activity against the promastigote forms of *L. amazonensis* | 40, 20, and 10 µg/ml for each metabolite | Amphotericin B | All spirocyclic erythrina-alkaloids showed low leishmanicidal activity.  Statistical analysis was not described. | [45] |
| *E. variegata* L. (Fabaceae) bark was collected in the regions of Zhejiang Province of China, in September 2016. | Stem bark was extracted three times with 95% alcohol under reflux for 3 hours. | *In vivo* in male Kunming mice *(Mus musculus*) | The anxiolytic activities were examined by using the elevated plus maze (EPM), light/dark box, and open field tests.  The forced swimming and tail suspension tests were performed to evaluate antidepressant activities. | 50, 100, and 200 mg/kg | Diazepam and fluoxetine | Significantly altered the levels of five neurotransmitters (dopamine, noradrenaline, serotonin, glutamate, and gamma-aminobutyric acid) in the brain tissue (p < 0.05).  The normally distributed data were subjected to two-way ANOVA followed by Dunnett's test. p<0.05 was considered statistically significant. | [46] |
| The fresh stem bark of *E. variegata* L. (Fabaceae) was collected in Jinghong, Yunnan Province, China, in January 2016. | The dried stem bark in the shade was refluxed with 65% ethanol and prenylated isoflavonoids were isolated. | *In vivo* on virgin Sprague-Dawley specific-pathogen-free (SPF) female rats (*Rattus norvegicus*) at 3-months-old | Bone protective effect | 600 mg/kg BW | Premarin (conjugated estrogen) 130 μg/kg BW | The extract reversed ovariectomy-induced deterioration of bone mineral density and bone microarchitecture in the tibia of ovariectomized rats.  Differences were analyzed statistically with one-way ANOVA followed by Tukey’s post-hoc test using the GraphPad PRISM software. p < 0.05 was considered statistically significant. | [47] |
| *E. indica* Lam. or *E. variegata* Linn. (Fabaceae) fresh leaves were collected in Pallavaram, Chennai, India | The leaves were extracted in a Soxhlet extractor continuously with methanol (50%) for 72 hours at 30 °C. | *In vivo* on isoniazid and rifampicin-induced hepatotoxicity male Sprague–Dawley rats (*Rattus norvegicus*) | Antihepatotoxicity | 100 and 200 mg/kg BW for 28 days | Silymarin | Dose of 100 and 200 mg/kg significantly reversed the elevation of transaminases and ALP activities toward normal (p < 0.05). Serum bilirubin and LDH were significantly reduced by these doses compared to hepatotoxic group rats (p < 0.05).  Data were subjected to one-way ANOVA using GraphPad Prism version 3.0. The variance in a set of data has been estimated by the Tukey multiple compare test. The values of p < 0.05 were considered statistically significant. | [48] |
| *E. falcata* Benth. (Fabaceae) stem bark was collected in October 2009 in Colombo, Paraná, Brazil. | Stem bark was extracted by percolation with EtOH/H_2_O (70:30) at room temperature for 1 month. | *In vivo* on male Wistar rats (*Rattus norvegicus*) | Modulators of fear memory | Vicenin-2 (0.1 mg/kg, 0.3 mg/kg, 1.0 mg/kg and 10.0 mg/kg)  Vitexin (0.1 mg/kg and 0.25 mg/kg)  Isovitexin (0.1 mg/kg and 0.25 mg/kg)  6-C-glycoside-diosmetin (0.1 mg/kg)  Fraction flavonoidic/Ff (0.65 mg/kg FfB and 0.90 mg/kg FfA) | Diazepam 4 mg/kg BW | Vitexin, isovitexin, diosmetin-6-C-glucoside metabolites, and flavonoid fractions resulted in significant retention of fear memory (p < 0.05) but did not prevent the extinction of fear memory.  Data were analyzed using one-way ANOVA followed by Bonferroni’s Multiple Comparison Test. Comparisons among dL-mean values were analyzed by two-way ANOVA followed by Dunnett’s Multiple Comparison. All statistical analyses were performed using the GraphPad Prism statistics program. Differences of the P < 0.05 level were considered statistically significant. | [49] |
| *E. variegata* L. (Fabaceae) stem bark was collected in Hoa Binh Province, Vietnam, in 2010. | The dried stem bark was extracted with 96% ethanol at room temperature for 2 weeks. | *In vitro* on:   - recombinant Aurora A - Aurora B - Aurora A domain - histone H3 proteins expressed as N-terminal His6-tagged fusion proteins in *E. coli* | Aurora kinase inhibitor | 15 to 238 µM | Not described | Derrone was isolated from the extract.  Derrone inhibited the phosphorylation of histone H3 at Ser10 both in the kinase assay and at the cellular level.  Derrone showed the most effective inhibition of the Aurora kinase domain (80%) at the concentration of 60 µM and exhibited higher activity against Aurora B than against Aurora kinase A, with IC_50_ values of 6 and 22.3 µM, respectively.  Statistical analyses were performed with GraphPad Prism 5. A value of p < 0.05 was considered statistically significant. | [50] |
| *E. abyssinica* Lam. ex DC. (Fabaceae) stem bark was collected in Embu County, Kenya, Africa. | The stem bark was extracted sequentially with dichloromethane and methanol | *In vitro* on chloroquine-sensitive (D6) and chloroquine resistance (W2) strains of *P. falciparum* | Antiplasmodial | 200 μg/ml (100%) to 3.125 μg/ml (1.56%) | Chloroquine | The dichloromethane extract revealed high antiplasmodial activity (IC_50_ ≤ 10 μg/ml) | [51] |
|  |  | *In vivo* on male Swiss albino mice (*Mus musculus*) |  | 100 mg/kg BW |  | The dichloromethane extract revealed high antiplasmodial activity (% suppression of 36.85 ± 5.07).  One-way ANOVA was used to determine differences between groups followed by Tukey’s post-hoc test for pairwise comparison and separation of means at p < 0.05. Unpaired student-test statistics were performed to determine differences in longevity. |  |
| *E. subumbrans* (Hassk.) Merr. (Fabaceae) stems collected in Thailand. | The stems were extracted with hexane and CH_2_Cl_2_. | *In vitro* on *P. falciparum* | Antiplasmodial | Not described | Not described | 5-hydroxysophoranone exhibited the highest antiplasmodial activity against *P. falciparum* (IC_50_ of 2.5 µg/mL).  Statistical analysis was not described. | [52] |
| *E. verna* Vell. or *E. mulungu* Mart. ex Benth. (Fabaceae) leaves and bark were collected in Rifaina, São Paulo State, Brazil. | Leaves and bark were extracted with a hydro-alcoholic solvent. | *In vitro* on the hippocampal cells from the fetuses of female Wistar rats (*Rattus norvegicus*) at 18 to 20 days of gestation. | Inhibitor of neuronal nicotinic receptors | Not described | Not described | The crude extracts and the alkaloids inhibited CNS nicotinic acetylcholine receptors, particularly the α4β2 subtype.  Statistical analysis was not described. | [53] |
| The various parts of *E. lysistemon* Hutch., Kew Bull. (Fabaceae) were collected between July 2001 and May 2002 in Gaborone, Botswana, Africa. | The twigs were extracted with ethyl acetate in vacuo while the leaves were with chloroform-methanol. | *In vitro* on brine shrimp (*Artemia salina*) larvae | Cytotoxicity | Not described | Ascorbic acid | The pterocarpans showed higher activities against the shrimps than those of the standards used. The isoflavones also showed relatively high toxicity with LD_50_ values lower than 25 ppm.  Statistical analysis was not described. | [54] |
