# Supplementary material for: Pharmacology activity, toxicity, and clinical trials of Erythrina genus plants (Fabaceae): an evidence-based review
Source: Front Pharmacol. 2023 Nov 16;14:1281150. doi: 10.3389/fphar.2023.1281150 (PMC10690608; doi:10.3389/fphar.2023.1281150)
Supplement: Supplementary file 2 [file Table2.docx]

**Table 2.** Studies of Metabolites Isolated from Erythrina Genus Plants on Different Pharmacological Assays

| Name of Metabolite isolated from Erythrina Genus Plants | Chemical Structure  (Molecular Formula; PubChem CID) | Pharmacological Assay Method | Reference |  |
| --- | --- | --- | --- | --- |
| **Flavonoids** | | | |  |
| 5-Hydroxysophoranone | 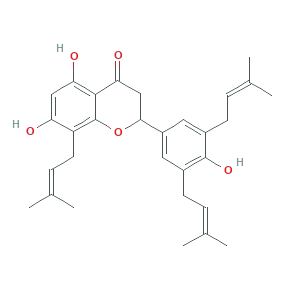  (C_30_H_36_O_5_; PubChem CID 42607927) | *In vitro* α-glucosidase inhibitory assay using acarbose as a positive control. | [5] |  |
|  |  | *In vitro* glycation inhibitory assay using quercetin as a positive control. | [5] |  |
|  |  | *In vitro* antiplasmodial activity against *P. falciparum* | [52] |  |
| 4′-hydroxy-6,3′,5′-triprenylisoflavanone | The chemical structure is not available in PubChem. | *In vitro* glycation inhibitory assay using quercetin as a positive control. | [5] |  |
|  |  | *In vitro* α-glucosidase inhibitory assay using acarbose as a positive control. | [5] |  |
| Pinocembrin | 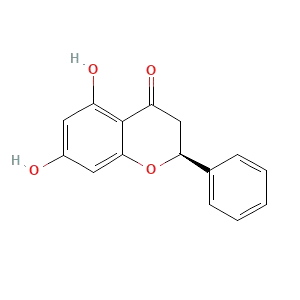  (C_15_H_12_O_4_; PubChem CID 68071) | *In vitro* α-glucosidase inhibitory assay using acarbose as a positive control. | [5] |  |
|  |  | *In vitro* glycation inhibitory assay using quercetin as a positive control. | [5] |  |
| 3,6,4′-trihydroxy flavone | The chemical structure is not available in PubChem. | *In vitro* α-glucosidase inhibitory assay using acarbose as a positive control. | [5] |  |
|  |  | *In vitro* glycation inhibitory assay using quercetin as a positive control. | [5] |  |
| 3,6-dihydroxyflavone | 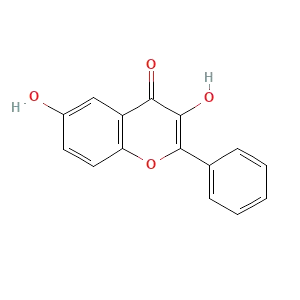  (C_15_H_10_O_4_; PubChem CID 688659) | *In vitro* α-glucosidase inhibitory assay using acarbose as a positive control. | [5] |  |
|  |  | *In vitro* glycation inhibitory assay using quercetin as a positive control. | [5] |  |
| 5-methoxy-7-hydroxy flavone | The chemical structure is not available in PubChem. | *In vitro* α-glucosidase inhibitory assay using acarbose as a positive control. | [5] |  |
|  |  | *In vitro* glycation inhibitory assay using quercetin as a positive control. | [5] |  |
| Lupalbigenin | 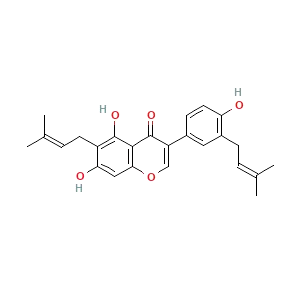  (C_25_H_26_O_5_; PubChem CID 10001388) | *In vitro* α-glucosidase inhibitory assay using acarbose as a positive control. | [5] |  |
|  |  | *In vitro* glycation inhibitory assay using quercetin as a positive control. | [5] |  |
| Erysubin F | 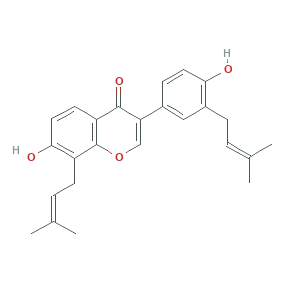  (C_25_H_26_O_4_; PubChem CID 12051847) | *In vitro* α-glucosidase inhibitory assay using acarbose as a positive control. | [5] |  |
|  |  | *In vitro* glycation inhibitory assay using quercetin as a positive control. | [5] |  |
| Genistein | 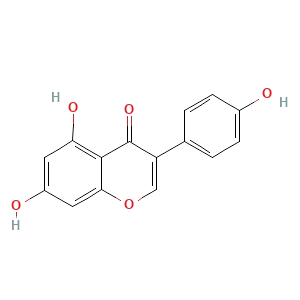  (C_15_H_10_O_5_; PubChem CID 5280961) | *In vitro* α-glucosidase inhibitory assay using acarbose as a positive control. | [5] |  |
|  |  | *In vitro* glycation inhibitory assay using quercetin as a positive control. | [5] |  |
|  |  | Estrogenic activity on 1-month-old immature female CD-1 mice *(Mus musculus*) | [42] |  |
| 8-prenyl genistein | The chemical structure is not available in PubChem. | Estrogenic activity on 1-month-old immature female CD-1 mice *(Mus musculus*) | [42] |  |
| 7,2′,4′-trihydroxy-8,3′,5′-(3″-methyl-but-2″-enyl) flavanone | The chemical structure is not available in PubChem. | *In vitro* α-glucosidase inhibitory assay using acarbose as a positive control. | [5] |  |
|  |  | *In vitro* glycation inhibitory assay using quercetin as a positive control. | [5] |  |
| Abyssinone V | 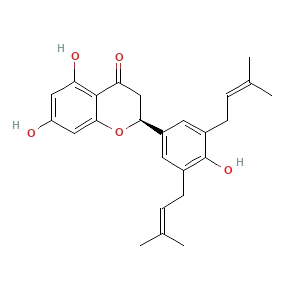  (C_25_H_28_O_5_; PubChem CID 442153) | *In vitro* α-glucosidase inhibitory assay using acarbose as a positive control. | [5] |  |
|  |  | *In vitro* glycation inhibitory assay using quercetin as a positive control. | [5] |  |
| Abyssinone V-4′ methyl ether | 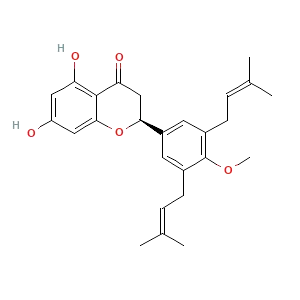  (C_26_H_30_O_5_; PubChem CID 6548074) | Antibacterial activity on Gram-negative and Gram-positive strains. | [18] |  |
| Eryzerin C | 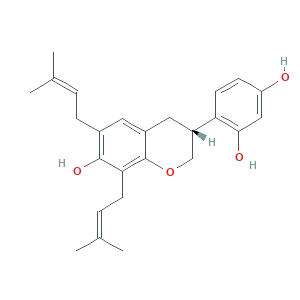  (C_25_H_30_O_4_; PubChem CID 10092034) | Antibacterial activity on Gram-negative and Gram-positive strains. | [18] |  |
| Lysisteisoflavone | 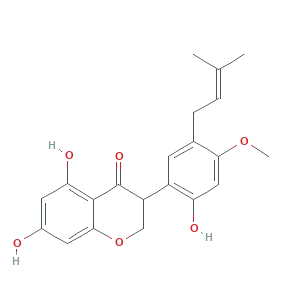  (C_21_H_22_O_6_; PubChem CID 5319135) | Antibacterial activity on Gram-negative and Gram-positive strains. | [18] |  |
| Wighteone (synonym = erythrinin B) | 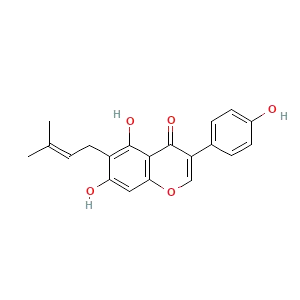  (C_20_H_18_O_5_; PubChem CID 5281814) | Antidiabetic and antiobesity by inhibiting PTP1B activity | [31] |  |
| Alpumisoflavone | 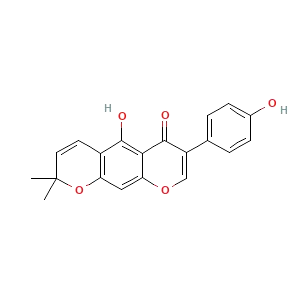  (C_20_H_16_O_5;_ PubChem CID 5490139) | Antibacterial activity on Gram-negative and Gram-positive strains. | [18] |  |
|  |  | Anticancer activity on human leukemia HL-60 cells | [24] |  |
|  |  | Anticancer activity on human lung cancer cell lines H2108 and H1299 | [25] |  |
| 4'-methoxy licoflavanone | The chemical structure is not available in PubChem. | Anticancer activity on human leukemia HL-60 cells | [24] |  |
| Licoflavanone-4’-O-methyl ether | | The chemical structure is not available in PubChem. | Antidiabetic and antiobesity by inhibiting PTP1B activity | [31] |
| 2’,7-dihydroxy-4’-methoxy-5’-(3-methyl but-2-enyl) isoflavone | | The chemical structure is not available in PubChem. | Antidiabetic and antiobesity by inhibiting PTP1B activity | [31] |
| Parisoflavone B | | The chemical structure is not available in PubChem. | Antidiabetic and antiobesity by inhibiting PTP1B activity | [31] |
| Derrone | | 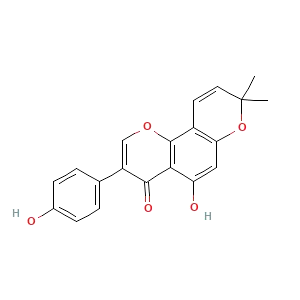  (C_20_H_16_O_5;_ PubChem CID 14704457) | Aurora kinase inhibitor on recombinant Aurora A, Aurora B, Aurora A domain, and histone H3 proteins expressed as N-terminal His6-tagged fusion proteins in *E. coli* | [50] |
|  | | | |  |
| **Pterocarpans** | | | |  |
| Eryvarin E | 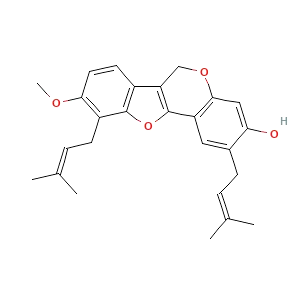  (C_26_H_28_O_4_; PubChem CID 15546809) | *In vitro* α-glucosidase inhibitory assay using acarbose as a positive control. | [5] |  |
|  |  | *In vitro* glycation inhibitory assay using quercetin as a positive control. | [5] |  |
| Coumestrol | 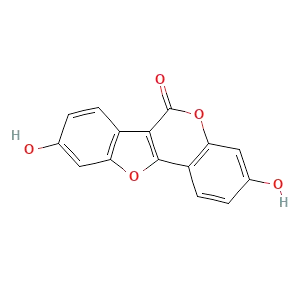  (C_15_H_8_O_5_; PubChem CID 5281707) | *In vitro* α-glucosidase inhibitory assay using acarbose as a positive control. | [5] |  |
|  |  | *In vitro* glycation inhibitory assay using quercetin as a positive control. | [5] |  |
| Eryvarin D | 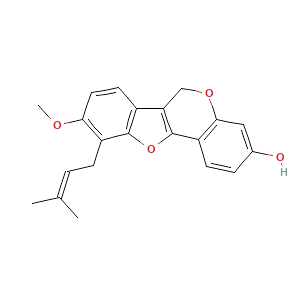  (C_21_H_20_O_4_; PubChem CID 15546808) | *In vitro* α-glucosidase inhibitory assay using acarbose as a positive control. | [5] |  |
|  |  | *In vitro* glycation inhibitory assay using quercetin as a positive control. | [5] |  |
| 1-methoxyerythrabyssin II | 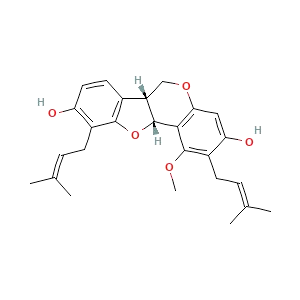  (C_26_H_30_O_5_; PubChem CID 24761044) | *In vitro* α-glucosidase inhibitory assay using acarbose as a positive control. | [5] |  |
|  |  | *In vitro* glycation inhibitory assay using quercetin as a positive control. | [5] |  |
| Erythrabyssin II | 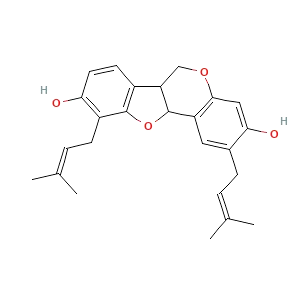  (C_25_H_28_O_4_; PubChem CID 5086400) | *In vitro* α-glucosidase inhibitory assay using acarbose as a positive control. | [5] |  |
|  |  | *In vitro* glycation inhibitory assay using quercetin as a positive control. | [5] |  |
| Erycristagallin | 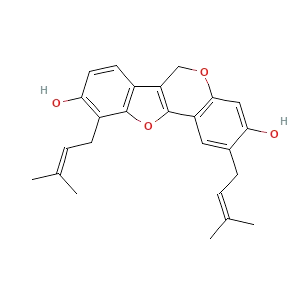  (C_25_H_26_O_4_; PubChem CID 10362969) | *In vitro* α-glucosidase and α-amylase inhibitory assay using acarbose as a positive control. | [5] |  |
|  |  | *In vitro* glycation inhibitory assay using quercetin as a positive control. | [5] |  |
| Erythribyssin N | 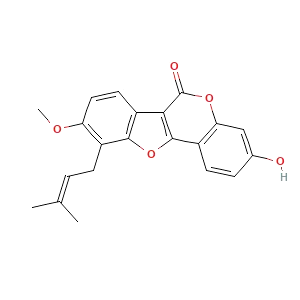  (C_21_H_18_O_5_; PubChem CID 46210318) | *In vitro* α-glucosidase and α-amylase inhibitory assay using acarbose as a positive control. | [5] |  |
|  |  | *In vitro* glycation inhibitory assay using quercetin as a positive control. | [5] |  |
| Erycristin | 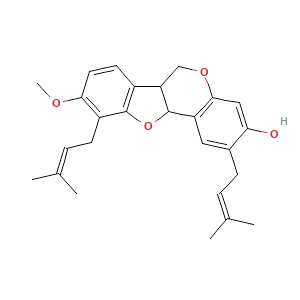  (C_26_H_30_O_4_; PubChem CID 13994537) | *In vitro* α-glucosidase and α-amylase inhibitory assay using acarbose as a positive control. | [5] |  |
|  |  | *In vitro* glycation inhibitory assay using quercetin as a positive control. | [5] |  |
| Sandwicensin | 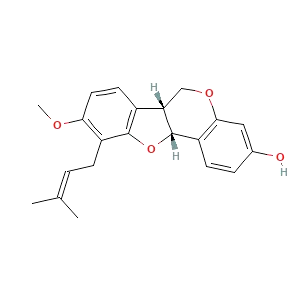  (C_21_H_22_O_4_; PubChem CID 467498) | *In vitro* α-glucosidase and α-amylase inhibitory assay using acarbose as a positive control | [5] |  |
|  |  | *In vitro* glycation inhibitory assay using quercetin as a positive control. | [5] |  |
| Gangetin | 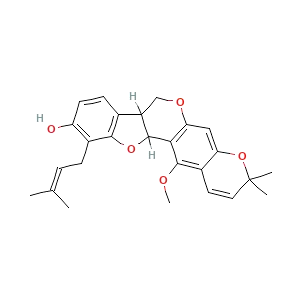  (C_26_H_28_O_5_; PubChem CID 317611) | *In vitro* α-glucosidase and α-amylase inhibitory assay using acarbose as a positive control | [5] |  |
|  |  | *In vitro* glycation inhibitory assay using quercetin as a positive control. | [5] |  |
| Erypoegin J | 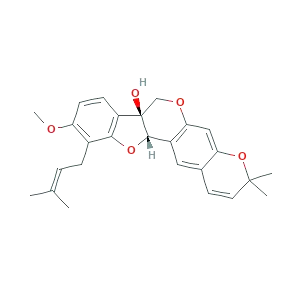  (C_26_H_28_O_5_; PubChem CID 11133568) | *In vitro* α-glucosidase and α-amylase inhibitory assay using acarbose as a positive control | [5] |  |
|  |  | *In vitro* glycation inhibitory assay using quercetin as a positive control. | [5] |  |
| Erybraedin A | 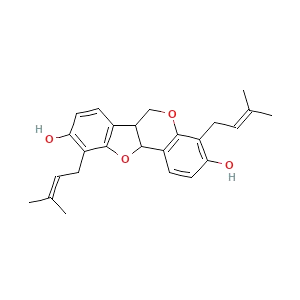  (C_25_H_28_O_4_; PubChem CID 362562) | Antibacterial activity on Gram-negative and Gram-positive strains. | [18] |  |
| Phaseollidin | 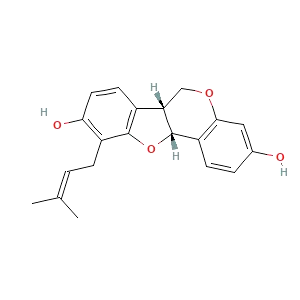  (C_20_H_20_O_4_; PubChem CID 119268) | *In vitro* α-glucosidase and α-amylase inhibitory assay using acarbose as a positive control | [5] |  |
|  |  | Antibacterial activity on Gram-negative and Gram-positive strains. | [18] |  |
|  |  | Cytotoxicity activity using brine shrimp lethal toxicity assay | [54] |  |
| Orientanol C | 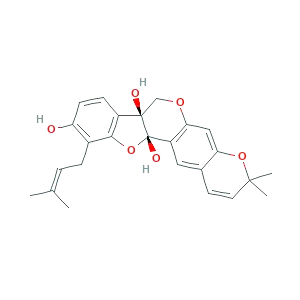  (C_25_H_26_O_6_; PubChem CID 42607512) | Anticancer activity on H4IIE hepatoma cells. | [26] |  |
|  |  | *In vitro* α-glucosidase and α-amylase inhibitory assay using acarbose as a positive control | [5] |  |
| Cristacarpin | 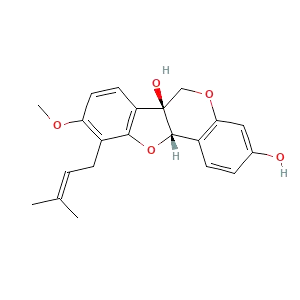  (C_21_H_22_O_5_; PubChem CID 126540) | Antibacterial activity on Gram-negative and Gram-positive strains. | [18] |  |
|  |  | Anticancer activity on H4IIE hepatoma cells. | [26] |  |
| Calopocarpin | 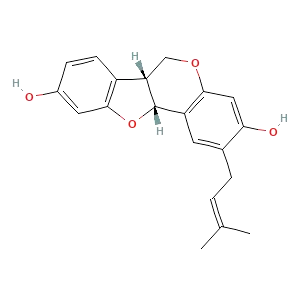  (C_20_H_20_O_4_; PubChem CID 11709595) | Anticancer activity on H4IIE hepatoma cells. | [26] |  |
| Isoneorautenol | 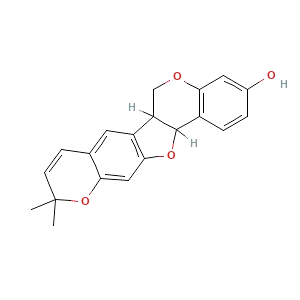  (C_20_H_18_O_4_; PubChem CID 73649) | Anticancer activity on H4IIE hepatoma cells. | [26] |  |
| Neorautenol | 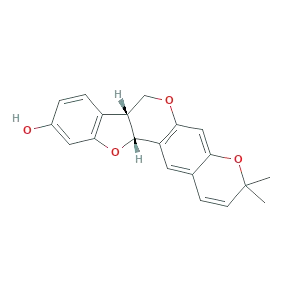  (C_20_H_18_O_4_; PubChem CID 11500744) | Anticancer activity on H4IIE hepatoma cells. | [26] |  |
| Phaseollin | 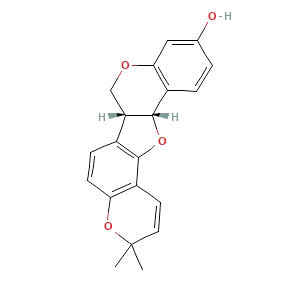  (C_20_H_18_O_4_; PubChem CID 91572) | Anticancer activity on H4IIE hepatoma cells. | [26] |  |
|  | | | |  |
| **Alkaloids** | | | |  |
| Hydroxyerysotrine | 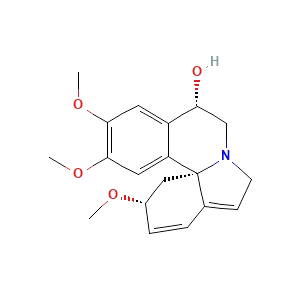  (C_19_H_23_NO_4_; PubChem CID 91572) | Anticancer activity on human leukemia HL-60 cells | [24] |  |
| Erythraline | 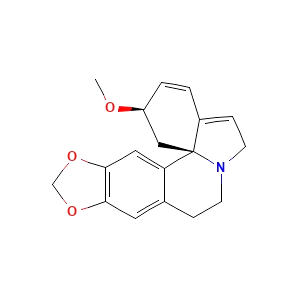  (C_18_H_19_NO_3_; PubChem CID 5317205) | Anti-inflammatory by inhibiting NO production on RAW264.7 cells | [41] |  |
|  |  | Leishmanicidal activity against the promastigote forms of *L. amazonensis* obtained from infected mice | [45] |  |
|  |  | Curare-like action on frogs | [55] |  |
| Erythrinine | 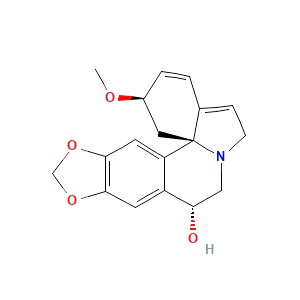  (C_18_H_19_NO_4_; PubChem CID 3084503) | Anti-inflammatory by inhibiting NO production on RAW264.7 cells | [41] |  |
| Hypaphorine | 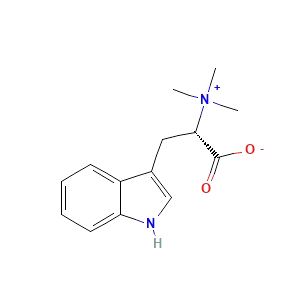  (C_14_H_19_N_2_O_2_; PubChem CID 442106) | Anti-inflammatory by inhibiting NO production on RAW264.7 cells | [41] |  |
| Erythramine | 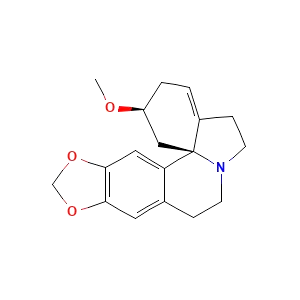  (C_18_H_21_NO_3_; PubChem CID 101289752) | Curare-like action on frogs | [55] |  |
| Erythratine | 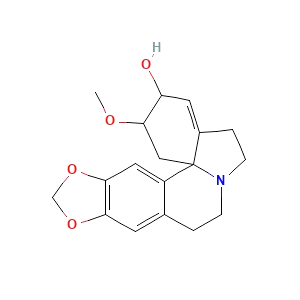  (C_18_H_21_NO_4_; PubChem CID 617879) | Curare-like action on frogs | [55] |  |
| Beta-erythroidine | 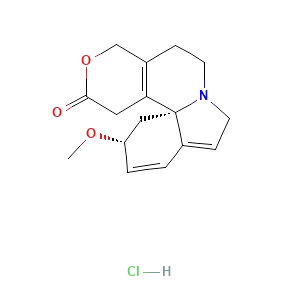  (C_16_H_20_ClNO_3_; PubChem CID 54601205) | Curare-like action on frogs | [55] |  |
|  | | | |  |
| **Glycosides** | | | |  |
| Vitexin | 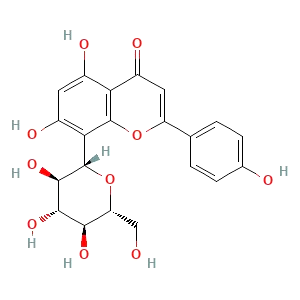  (C_21_H_20_O_10_; PubChem CID 5280441) | Modulator of fear memory on male Wistar rats (*Rattus norvegicus*) | [49] |  |
| Isovitexin | 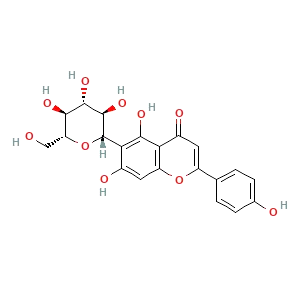  (C_21_H_20_O_10_; PubChem CID 162350) | Modulator of fear memory on male Wistar rats (*Rattus norvegicus*) | [49] |  |
| Diosmetin-6-C-glucoside | 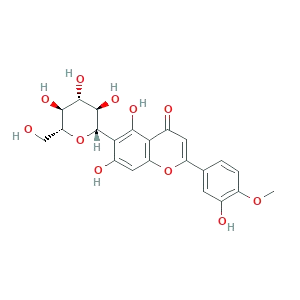  (C_22_H_22_O_11_; PubChem CID 162350) | Modulator of fear memory on male Wistar rats (*Rattus norvegicus*) | [49] |  |
| Vicenin 2 | 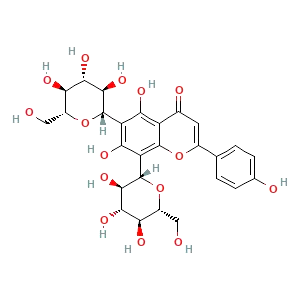  (C_27_H_30_O_15_; PubChem CID 442664) | Modulator of fear memory on male Wistar rats (*Rattus norvegicus*) | [49] |  |
|  | | | |  |
| **Coumarins** | | | |  |
| Xanthoxyletin | 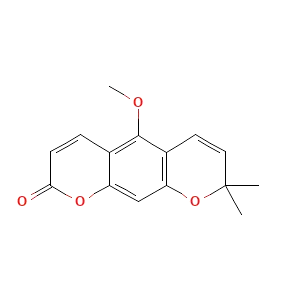  (C_15_H_14_O_4_; PubChem CID 66548) | Anticancer activity on SGC-7901 cells. | [27] |  |
|  | | | |  |
| **Triterpenoids** | | | |  |
| Sophoradiol | 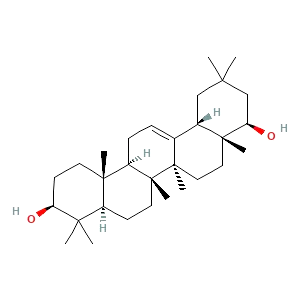  (C_30_H_50_O_2_; PubChem CID 9846221) | *In vitro* antiplasmodial activity against *P. falciparum* | [52] |  |
| Lupeol | 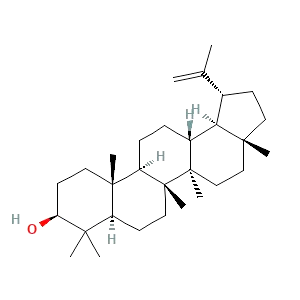  (C_30_H_50_O; PubChem CID 259846) | *In vitro* antiplasmodial activity against *P. falciparum* | [52] |  |
| Cycloeucalenol | The chemical structure is not available in PubChem. | *In vitro* antiplasmodial activity against *P. falciparum* | [52] |  |
| Melilotigenin C | 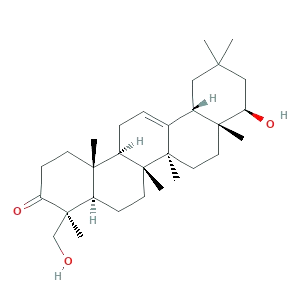  (C_30_H_48_O_3_; PubChem CID 10551785) | *In vitro* antiplasmodial activity against *P. falciparum* | [52] |  |
|  | | | |  |
| **Chromenes** | | | |  |
| 2,2-dimethyl-2H-chromene-6-carboxaldehyde | The chemical structure is not available in PubChem. | *In vitro* glycation inhibitory assay using quercetin as a positive control. | [5] |  |
|  |  | *In vitro* α-glucosidase inhibitory assay using acarbose as a positive control. | [5] |  |
|  | | | |  |
| **Steroids** | | | |  |
| Stigmast-4-en-3-one | 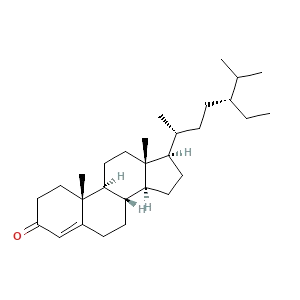  (C_29_H_48_O; PubChem CID 5484202) | *In vitro* antiplasmodial activity against *P. falciparum* | [52] |  |
| Stigmasta-4,22-dien-3-one | 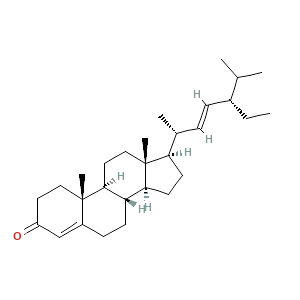  (C_29_H_46_O; PubChem CID 6442194) | *In vitro* antiplasmodial activity against *P. falciparum* | [52] |  |
| 3beta-hydroxystigmast-5-en-7-one | 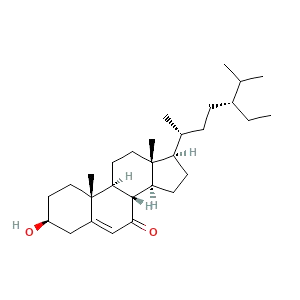  (C_29_H_48_O_2_; PubChem CID 160608) | *In vitro* antiplasmodial activity against *P. falciparum* | [52] |  |
| 3beta-hydroxystigmast-5,22-dien-7-one | The chemical structure is not available in PubChem. | *In vitro* antiplasmodial activity against *P. falciparum* | [52] |  |
|  | | | |  |
| **Carboxylic Acids** | | | |  |
| 2-hydroxy-4-methoxy-cinnamic acid | 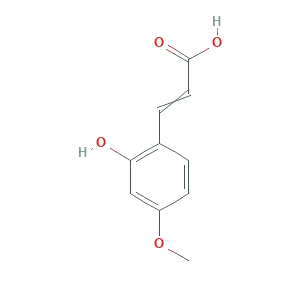  (C_10_H_10_O_4_; PubChem CID 20985633) | *In vitro* glycation inhibitory assay using quercetin as a positive control. | [5] |  |
|  |  | *In vitro* α-glucosidase inhibitory assay using acarbose as a positive control. | [5] |  |
| **Total = 63 metabolites** | | | |  |
